# Supplementary material for: Visualization and functional dissection of coaxial paired SpoIIIE channels across the sporulation septum
Source: eLife. 2015 May 7;4:e06474. doi: 10.7554/eLife.06474 (PMC4423119; doi:10.7554/eLife.06474)
Supplement: Supplementary file 1. — (A) Strains used in this study. (B) Plasmid used in this study. (C) Oligonucleotides used in this sudy. (D) Sequence of amino acid residues of the linkers within the SpoIIIE-GFP-SsrA*. (E) Spore titers of strains containing the different spoIIIE fusion proteins used in this study. DOI: http://dx.doi.org/10.7554/eLife.06474.030 [file elife-06474-supp1.docx]

| **Supplementary File 1 A. Strains used in this study** | | |
| --- | --- | --- |
| **Strain** | **Genotype or description** | **Reference, source or construction^a^** |
| PY79 | Wild type | (Youngman et al., 1984) |
| EBS606 | *cotC::cat::PspoIIQ-CFP*Ω*tet* | Becker and Pogliano, 2007 |
| JLG170 | *amyE*::*PspoIIQ-sspB*Ω*cat* | pJLG13→ PY79 (Cm^R^) |
| JLG180 | *thrC*::*PspoIID-sspB*Ω*spc* | pJLG20→ PY79 (Sp^R^) |
| JLG247 | *sigA-sfGFP-ssrA**Ω*kan* | pJLG49→ PY79 (Km^R^) |
| JLG259 | *thrC*::*PspoIID-sspB*Ω*spc sigA-sfGFP-ssrA**Ω*kan* | JLG247→ JLG180 (Km^R^) |
| JLG261 | *amyE*::*PspoIIQ-sspB*Ω*cat sigA-sfGFP-ssrA**Ω*kan* | JLG247→ JLG170 (Km^R^) |
| JLG323 | *amyE*::*PspoIIQ-sspB*Ω*cat thrC*::*PspoIID-sspB*Ω*spc* | JLG170→ JLG180 (Cm^R^) |
| JLG451 | *spoIIIE-sfGFP-ssrA**Ω*kan* | pJLG72→ PY79 (Km^R^) |
| JLG452 | *amyE*::*PspoIIQ-sspB*Ω*cat spoIIIE-sfGFP-ssrA**Ω*kan* | JLG451→ JLG170 (Km^R^) |
| JLG453 | *thrC*::*PspoIID-sspB*Ω*spc spoIIIE-sfGFP-ssrA**Ω*kan* | JLG451→ JLG180 (Km^R^) |
| JLG454 | *amyE*::*PspoIIQ-sspB*Ω*cat thrC*::*PspoIID-sspB*Ω*spc spoIIIE-sfGFP-ssrA**Ω*kan* | JLG451→ JLG323 (Km^R^) |
| JLG571 | *spoIID298 spoIIM::Tn917Ωmls spoIIP::tet spoIIIE-tdEOS2Ωkan* | TCF24→JLG559 (Km^R^) |
| JLG808 | *spoIIIE73-11(G467S)-sfGFP-ssrA**Ω*kan* | pJLG118→ KP541 (Km^R^) |
| JLG821 | *amyE*::*PspoIIQ-sspB*Ω*cat spoIIIE73-11(G467S)-sfGFP-ssrA**Ω*kan* | JLG808→ JLG170 (Km^R^) |
| JLG823 | *thrC*::*PspoIID-sspB*Ω*spc spoIIIE73-11-sfGFP-ssrA**Ω*kan* | JLG808→ JLG180 (Km^R^) |
| JLG825 | *amyE*::*PspoIIQ-sspB*Ω*cat thrC*::*PspoIID-sspB*Ω*spc spoIIIE73-11(G467S)-sfGFP-ssrA**Ω*kan* | JLG808→ JLG323 (Km^R^) |
| JLG917 | *gyrA-sfGFP-ssrAΩkan* | pJLG125→ PY79 (Km^R^) |
| JLG919 | *gyrA-sfGFP-ssrAΩkan amyE*::*PspoIIQ-sspB*Ω*cat* | JLG917→ JLG170 (Km^R^) |
| JLG978 | *amyE*::*PspoIIQ-sspB*Ω*cat cotC::cat::PspoIIQ-CFP*Ω*tet* | EBS606→ JLG170 (Tet^R^) |
| JLG979 | *thrC*::*PspoIID-sspB*Ω*spc cotC::cat::PspoIIQ-CFP*Ω*tet* | EBS606→ JLG180 (Tet^R^) |
| JLG980 | *amyE*::*PspoIIQ-sspB*Ω*cat thrC*::*PspoIID-sspB*Ω*spc cotC::cat::PspoIIQ-CFP*Ω*tet* | EBS606→ JLG323 (Tet^R^) |
| JLG981 | *spoIIIE-sfGFP-ssrA**Ω*kan cotC::cat::PspoIIQ-CFP*Ω*tet* | EBS606→ JLG451 (Tet^R^) |
| JLG1001 | *amyE*::*PspoIIQ-sspB*Ω*cat spoIIIE-sfGFP-ssrA**Ω*kan cotC::cat::PspoIIQ-CFP*Ω*tet* | EBS606→ JLG452 (Tet^R^) |
| JLG1002 | *thrC*::*PspoIID-sspB*Ω*spc spoIIIE-sfGFP-ssrA**Ω*kan cotC::cat::PspoIIQ-CFP*Ω*tet* | EBS606→ JLG453 (Tet^R^) |
| JLG1003 | *amyE*::*PspoIIQ-sspB*Ω*cat thrC*::*PspoIID-sspB*Ω*spc spoIIIE-sfGFP-ssrA**Ω*kan cotC::cat::PspoIIQ-CFP*Ω*tet* | EBS606→ JLG454 (Tet^R^) |
| JLG1281 | *gyrA-sfGFP-ssrAΩkan thrC::PspoIID-sspBΩspc* | JLG917→ JLG180 (Km^R^) |
| JS00 | *spoIIIE-dendra2*Ω*kan sigE*::*erm* | KP161→TCF25 (Em^R^) |
| JS03 | *spoIIIE-tdEOS*Ω*kan* *sigE*::*erm* | KP161→TCF24 (Em^R^) |
| JS04 | *spoIIIE73-11(G467S)-tdEOS*Ω*kan* *sigE*::*erm* | KP161→TCF27 (Em^R^) |
| KP92 | *spoIIIE36* | (Wu and Errington, 1994) |
| KP161 | *sigE*::*erm* | (Kenney and Moran, 1987) |
| KP541 | *spoIIIE^ATP-^ (*G467S; ATPase mutant) | (Sharp and Pogliano, 1999) |
| TCF24 | *spoIIIE-tdEOS*Ω*kan* | (Fleming et al., 2010) |
| TCF25 | *spoIIIE-dendra2*Ω*kan* | pTF25→ PY79 (Kan^R^) |

^a^ Plasmid or genomic DNA employed (right side the arrow) to transform an existing strain (left side the arrow) to create a new strain are listed. The drug resistance is noted in parentheses.

| **Supplementary File 1 B. Plasmid used in this study** | |
| --- | --- |
| **Plasmid** | **Description** |
| pTF25 | *spoIIIE-dendra2Ωkan* |
| pJLG3 | *ssrA*Ωkan* |
| pJLG7 | *thrC::sspB Ωspec* |
| pJLG13 | *amyE::P_spoIIQ_- sspB Ωcat* |
| pJLG20 | *thrC::P_spoIID_- sspB Ωspec* |
| pJLG36 | *sfGFP-ssrA*Ωkan* |
| pJLG38 | *sfGFPΩkan* |
| pJLG49 | *sigA-sfGFP-ssrA*Ωkan* |
| pJLG72 | *spoIIIE-sfGFP-ssrA*Ωkan* |
| pJLG112 | *gyrA-ssrA*Ωkan* |
| pJLG118 | *spoIIIE^ATP-^-sfGFP-ssrA*Ωkan* |
| pJLG125 | *gyrA-sfGFP-ssrA*Ωkan* |

| **Supplementary File 1 C. Oligonucleotides used in this sudy** | |
| --- | --- |
| **Primer** | **Sequence^a^** |
| TF-55 | *gggcactagt*ATGAACACCCCGGGAATTAACCTG |
| TF-56 | *cccactagtttatt*ACCACACCTGGCTGGGCAG |
| JLG-1 | *tttttgctagcgcagcaaatgatgaaaactattcagaaaattatgcacttggaggataa*aTGAGAGAGGAAGAAAAGGG |
| JLG-5 | *tttttctgcag*AATTGGGACAACTCCAGTG |
| JLG-7 | AATTGGGACAACTCCAGTG |
| JLG-16 | *tttttgaattcggatcc*ATGGATTTGTCACAGCTAACAC |
| JLG-17 | *tttttagatctgctagc*TTACTTCACAACGCGTAATGC |
| JLG-32 | *tttttcggccggctagc*TTACTTCACAACGCGTAATGC |
| JLG-33 | *tttttgcatgcgctagcagcgcaagcgcaagcgca*GCTAAAGGCGAAGAACTGTTTAC |
| JLG-34 | *tttttactagt*TTTATACAGTTCATCCATGCC |
| JLG-55 | TTTATACAGTTCATCCATGCC |
| JLG-77 | GCTAGCAGCGCAAGCGC |
| JLG-86 | P-TAAATGAGAGAGGAAGAAAACGG |
| JLG-87 | P-TCATTTATACAGTTCATCCATGCC |
| JLG-95 | CATGGATTACGCGTTAACCC |
| JLG-96 | GCACTTTTCGGGGAAATGTG |
| JLG-130 | *cactggagttgtcccaattc*GATGGAACGGGTCTTGAAG |
| JLG-131 | *cacatttccccgaaaagtgc*CCATTCGGTATGTACTCCGC |
| JLG-132 | *gggttaacgcgtaatccatg*AATGACCTAAGTGTACCGCC |
| JLG-133 | *cttgcgcttgcgctgctagc*TTCAAGGAAATCTTTCAAACG |
| JLG-184 | GCTAGCGCAGCAAATGATG |
| JLG-232 | *cactggagttgtcccaattc*AGGGAGTTCCGCTTTCTATAG |
| JLG-233 | *cacatttccccgaaaagtgc*ATGAAATCTGAATTTATCCGC |
| JLG-234 | *gggttaacgcgtaatccatg*GAGCTAAATGTCTACAACGGG |
| JLG-236 | *cttgcgcttgcgctgctagc*AGAAGAGAGCTCATCATATTTCTC |
| JLG-245 | GTTGTCGGACCGTATGAAGG |
| JLG-248 | CCTTCATACGGTCCGACAAC |
| JLG-416 | *gggttaacgcgtaatccatg*TATGGGAACAAACGAAGATG |
| JLG-417 | *catcatttgctgcgctagc*CACTTCTTCTTGTTCTTCTTCATTC |
| JLG-418 | *cactggagttgtcccaattc*AAAAAAGCGCAGCTGAAATAG |
| JLG-419 | *cacatttccccgaaaagtgc*GGCTTTCTGGTTAGGTACCG |
| JLG-450 | *gggttaacgcgtaatccatg*TTATCGCAATCTTGCAGCTG |
| JLG-539 | *ggcatggatgaactgtataaa*GCTAGCGCAGCAAATGATG |
| JLG-540 | *gcgcttgcgctgctagc*CACTTCTTCTTGTTCTTCTTCATTC |

^a^In capital letters are shown the regions of the primer that anneals to the template. Restriction sites and homology regions for Gibson assembly are shown in italics. A letter “P” before the sequence indicates that the primer is phosphorylated at the 5’end.

| **Supplementary File 1 D. Sequence of amino acid residues of the linkers within the SpoIIIE-GFP-SsrA*** | | |
| --- | --- | --- |
| **Linker between** | **Sequence** | **# of residues** |
| SpoIIIE and GFP | AlaSerSerAlaSerAlaSerAla | 8 |
| GFP and SsrA | ThrSer | 2 |

Spore titers were determined by heat kill assays. The average of three independent experiments is shown for every strain.

| **Supplementary File 1 E. Spore titers of strains containing the different *spoIIIE* fusion proteins used in this study.** | | |
| --- | --- | --- |
| **Strain** | **SpoIIIE fusion protein** | **Average spore titer** |
| PY79 | Wild type | 3.0x10^8^ |
| KP92 | *spoIIIE36* | 0.0x10^0^ |
| TCF24 | *spoIIIE-tdEOS* | 1.2 x10^8^ |
| TCF25 | *spoIIIE-dendra2* | 3.2 x10^8^ |
| JLG451 | *spoIIIE-sfGFP-ssrA** | 2.3 x10^8^ |

**References**

Becker EC, Pogliano K. 2007. Cell-specific SpoIIIE assembly and DNA translocation polarity are dictated by chromosome orientation. *Molecular Microbiology* **66:**1066–79. doi:10.1111/j.1365-2958.2007.05992.x.

Fleming TC, Shin JY, Lee S-H, Becker E, Huang KC, Bustamante C, Pogliano K. 2010. Dynamic SpoIIIE assembly mediates septal membrane fission during *Bacillus subtilis* sporulation. *Genes Dev* **24**:1160–72. doi:10.1101/gad.1925210.

Kenney TJ, Moran CP. 1987. Organization and regulation of an operon that encodes a sporulation-essential sigma factor in *Bacillus subtilis*. *Journal of Bacteriology* **169**:3329–39.

Sharp MD, Pogliano K. 1999. An in vivo membrane fusion assay implicates SpoIIIE in the final stages of engulfment during *Bacillus subtilis* sporulation. *Proc Natl Acad Sci USA* **96**:14553–58. doi:10.1073/pnas.96.25.14553.

Wu LJ, Errington J. 1994. *Bacillus subtilis* SpoIIIE protein required for DNA segregation during asymmetric cell division. *Science* **264**:572–5. doi:10.1126/science.8160014.

Youngman P, Perkins JB, Losick R. 1984. A novel method for the rapid cloning in *Escherichia coli* of *Bacillus subtilis* chromosomal DNA adjacent to Tn917 insertions. *Mol Gen Genet* **195**:424–33.
